# Extracallosal Structural Connectivity Is Positively Associated With Language Performance in Well-Performing Children Born Extremely Preterm

**DOI:** 10.3389/fped.2022.821121

**Published:** 2022-03-18

**Authors:** Maria E. Barnes-Davis, Brady J. Williamson, Stephanie L. Merhar, Usha D. Nagaraj, Nehal A. Parikh, Darren S. Kadis

**Affiliations:** ^1^Perinatal Institute, Cincinnati Children's Hospital Medical Center, Cincinnati, OH, United States; ^2^Department of Pediatrics, University of Cincinnati, Cincinnati, OH, United States; ^3^Department of Radiology, University of Cincinnati, Cincinnati, OH, United States; ^4^Pediatric Neuroimaging Research Consortium, Cincinnati Children's Hospital Medical Center, Cincinnati, OH, United States; ^5^Neurosciences and Mental Health, Hospital for Sick Children, Toronto, ON, Canada; ^6^Department of Physiology, University of Toronto, Toronto, ON, Canada

**Keywords:** prematurity, language, connectivity, magnetic resonance imaging (MRI), diffusion

## Abstract

Children born extremely preterm (<28 weeks gestation) are at risk for language delay or disorders. Decreased structural connectivity in preterm children has been associated with poor language outcome. Previously, we used multimodal imaging techniques to demonstrate that increased functional connectivity during a stories listening task was positively associated with language scores for preterm children. This functional connectivity was supported by extracallosal structural hyperconnectivity when compared to term-born children. Here, we attempt to validate this finding in a distinct cohort of well-performing extremely preterm children (EPT, *n* = 16) vs. term comparisons (TC, *n* = 28) and also compare this to structural connectivity in a group of extremely preterm children with a history of language delay or disorder (EPT-HLD, *n* = 8). All participants are 4–6 years of age. We perform q-space diffeomorphic reconstruction and functionally-constrained structural connectometry (based on fMRI activation), including a novel extension enabling between-groups comparisons with non-parametric ANOVA. There were no significant differences between groups in age, sex, race, ethnicity, parental education, family income, or language scores. For EPT, tracks positively associated with language scores included the bilateral posterior inferior fronto-occipital fasciculi and bilateral cerebellar peduncles and additional cerebellar white matter. Quantitative anisotropy in these pathways accounted for 55% of the variance in standardized language scores for the EPT group specifically. Future work will expand this cohort and follow longitudinally to investigate the impact of environmental factors on developing language networks and resiliency in the preterm brain.

## Introduction

Prematurity has long been associated with brain injury, maturational abnormalities, and cognitive impairment (1, 2). Brain injury has been reported across countries and continents, including classic findings on cranial ultrasound such as periventricular leukomalacia (PVL) and intraventricular hemorrhage (IVH); other diagnoses based on magnetic resonance imaging (MRI) such as white matter injury of prematurity (3); and newer, objectively determined entities such as diffuse excessive high signal intensity (DEHSI) (4) which is currently referred to as diffuse white matter abnormality (DWMA) (5, 6). Even in the absence of overt brain injury/immaturity diagnosed by clinical imaging, risk for neurodevelopmental deficits persist. These deficits include language delays and disorders, cognitive delays and intellectual disability, academic and behavioral difficulties, motor delays, and cerebral palsy (1, 2, 7–13). While survival is improving for children born extremely preterm (EPT, <28 weeks of completed gestation), particularly those born in the “periviable” period below 25 weeks completed gestation, rates of moderate to severe neurodevelopmental impairment are not improving (1, 2). This is important because prematurity rates are on the rise again, impacting 10% of children born in the United States and globally (14, 15). Thus, the neurodevelopmental sequelae of prematurity represent a significant public health crisis.

The majority of neuroimaging studies investigating development of the preterm brain rely on imaging obtained in infancy or in adulthood (16). There is a need for neuroimaging research studying children born EPT through critical periods of development, as neurodevelopmental scores at 2 years of age or earlier poorly predict skills in later childhood (17). This is especially true regarding the development of language. Later language skills have proven harder to predict (vs. cognitive and motor outcome) on the basis of term equivalent imaging or early behavioral testing (such as a Bayley Scales of Infant and Toddler Development III assessment at 2 years corrected age) (18–20). EPT children are at increased risk for delayed or disordered language (2, 7, 9, 18, 21, 22). Known sequelae of prematurity include decreased college attendance, decreased professional attainments, increased risk for abuse and non-accidental trauma, decreased likelihood of developing significant romantic partnerships in adulthood, decreased likelihood of having their own children in adulthood, and decreased quality of life (23–27). Language is important for general cognitive and scholastic development and establishment of relationships with both caregivers and peers (28).

We are studying a unique cohort of 4–6-year-old children born extremely preterm and their term developmental comparisons (TC) with multimodal neuroimaging, including magnetoencephalography (MEG), structural MRI, task-based and resting-state functional magnetic resonance imaging (fMRI), and multi-shell high resolution diffusion weighted imaging (dMRI) (29–32). Our overarching aim is to investigate brain-based markers of resiliency in preterm children, particularly for language and cognitive outcomes. Previously, we reported increased interhemispheric functional (right-to-left bitemporal) connectivity in well-performing EPT children vs. TC using MEG (29). Diffusion imaging obtained at the same study visit revealed that increased functional connectivity is supported by increased structural connectivity in extracallosal pathways, including cerebellar white matter, and positively correlated with language performance (31). In this current study, we aim to externally validate this finding and compare structural connectivity profiles in well-performing extremely preterm children (the EPT group) with TC and extremely preterm children with a history of being diagnosed with language delay, disorder, or impairment (the EPT-HLD group). We will determine the extent to which extracallosal connectivity is a marker of resiliency for language in this cohort. To that end, we will test the following hypotheses:

1. Well-performing children born EPT will have increased extracallosal structural connectivity vs. TC children at 4–6 years of age.
2. Children born EPT-HLD will have lower scores on standardized language assessments than their well-performing EPT counterparts and will lack this extracallosal hyperconnectivity.
3. Extracallosal structural connectivity, including that of cerebellar white matter, will be positively associated with language scores in all children born preterm, but not for the TC group.

## Materials and Methods

### Study Design and Participants

This is a multimodal neuroimaging study of a cohort consisting of 69 children (36 TC, 19 EPT, 14 EPT-HLD) aged from 4 to <7 years recruited and tested at Cincinnati Children's Hospital Medical Center in the United States from 2018 to 2021. These participants are distinct from our pilot study conducted in 2015-2016. 19 children were enrolled in our well-performing EPT group and had no known neurological disorders (such as cerebral palsy, autism, or ADHD) or known prior brain injury (such as PVL or moderate to severe IVH) and had no known speech, language, or learning disorders. 14 children were enrolled in our group of EPT-HLD children, meaning that they had no known neurological disorders or brain injury, but did have a history of language disorder or delay. To be included in the EPT-HLD group, the diagnosis needed to be reported by the parent or pediatrician and confirmed by a speech language pathologist (SLP) in their electronic medical record. All children in the EPT-HLD group had a documented history of therapy from a SLP. 36 children were enrolled in our TC group, meaning they were born full term (defined here as 37 weeks or more completed gestation) and had no known neurological disorder, brain injury, or speech, language, or learning disorders or history of therapy for such a disorder. All groups met standard MRI safety exclusion criteria. Full inclusion and exclusion criteria are detailed in Table 1. Children for both preterm groups were recruited from Cincinnati-area neonatal intensive care units (NICU) and follow-up clinics. Children for the TC group were recruited from area pediatric clinics and community-wide research advertisements. Informed written consent was obtained from the parent or legal guardian of all participants and verbal assent was obtained from the child. This study was approved by the institutional review boards (IRB) of Cincinnati Children's Hospital Medical Center, the Perinatal Scientific Review Committee of the TriHealth medical system, and the University of Cincinnati. The study was performed in accordance with the Declaration of Helsinki and the US Federal Policy for the Protection of Human Subjects. Assessments were completed during a single visit and required a total of 4 h of participation. Of the 69 children enrolled, 17 children were excluded from the final analyses (8 TC, 6 EPT-HLD, and 3 EPT) due to incidental findings on structural imaging (5 participants), incomplete or corrupted data sets (10 participants), or poor quality images (2 participants). Thus, the final sample size included in subsequent analyses is 52 participants (28 TC, 8 EPT-HLD, 16 EPT).

**Table 1:** Inclusion and exclusion criteria.

**Term comparison children (TC)**
Age 4.0 to <7 years
Personal history of term birth with gestational age of 37 weeks to 42 weeks
Informed consent of parent, assent of children
Negative for
Cerebral palsy
IVH Grade III or IV or parenchymal lesion/bleed on cranial ultrasound
Seizures
Migraines
History of speech, language, or learning disability
History of other neurologic or psychiatric disease, such as autism or ADHD
Standard MRI exclusion criteria, including orthodontic braces or metallic implants/devices
**Extremely preterm children without diagnosis of language delay (EPT)**
Age 4.0 to <7 years
Personal history of preterm birth with gestational age of <28 weeks
Personal history of birth weight <1,500 grams
Informed consent of parent, assent of children
Negative for
Cerebral palsy
IVH Grade III or IV or parenchymal lesion/bleed on cranial ultrasound
Seizures
Migraines
History of speech, language, or learning disability
History of other neurologic or psychiatric disease, such as autism or ADHD
Standard MRI exclusion criteria, including orthodontic braces or metallic implants/devices
**Extremely preterm children with history of language delay/disorder/deficit (EPT-HLD)**
Age 4.0 to <7 years
Personal history of preterm birth with gestational age of <28 weeks
Personal history of birth weight <1,500 grams
Personal history of language delay, disorder, or deficit
(Defined as current or prior formal diagnosis by pediatrician and/or speech language pathologist of language delay, deficit, disorder, or impairment in the medical record or history of speech/language therapy for such diagnosis)
Informed consent of parent, assent of children
Negative for
Cerebral palsy
IVH Grade III or IV or parenchymal lesion/bleed on cranial ultrasound
Seizures
Migraines
History of other neurologic or psychiatric disease, such as autism or ADHD
Standard MRI exclusion criteria, including orthodontic braces or metallic implants/devices

### Demographic and Neuropsychological Assessments

Upon arrival to our institution, informed consent was obtained by study personnel. Children then underwent testing of language and general abilities with the Peabody Picture Vocabulary Test (PPVT4) (33); Expressive Vocabulary Test (EVT2) (34); the Wechsler Nonverbal Scale of Ability (WNV) (35), and the Word Structure subtest of the Clinical Evaluation of Language Fundamentals: Preschool Edition (CELF-P) (36). The PPVT4 and EVT2 have high validity and reliability, correlate highly with verbal intelligence in children, can be used across the lifespan to provide a robust assay of language abilities, and are widely used in studies of prematurity (37–43). The WNV is a non-verbal assessment of general abilities (related to intelligence, or IQ) specifically designed for use in populations for whom English is a second language or who are at increased risk for language difficulties. In addition to our two receptive and expressive vocabulary assessments, the Word Structure subtest of the CELF-P was used as a non-vocabulary language task, assessing language pragmatics and morphology. While children were participating in this testing battery, parents were completing forms on demographics, medical history, family income and education, and handedness (the Edinburgh Handedness Inventory) (44).

### Multimodal Neuroimaging Acquisition

#### Structural MRI Acquisition and Preprocessing

All MR scanning was conducted on a Philips Achieva 3.0T scanner. 3D T1- and T2-weighted structural images had 1.0 x 1.0 x 1.0 mm isotropic voxels with a 256 x 256 resolution matrix. T2 scans had a repetition time and echo time (TR/TE) = 2500 ms / 3.68 ms and lasted 4 min and 28 s. T1 scans had TR/TE = 8.1 ms / 3.7 ms and lasted 5 min and 15 s. All structural preprocessing was performed in AFNI (45). All scans were read by a board-certified radiologist with certificates of added qualifications in pediatric radiology and neuroradiology (UN).

#### Functional MRI Acquisition and Joint Activation Map

Task-based fMRI recordings were obtained while children participated in a widely used passive stories listening paradigm (29, 46). This involved multi-echo acquisition (TE 14/32/50 ms, TR 1226.45 ms), acquired with multiband (factor 3) and in-plane SENSE (factor 3) acceleration. The multi-echo acquisition provides the ability to isolate BOLD signal through independent component analysis (47, 48). Functional imaging voxels were 3.0 x 3.0 x 3.0 mm. Functional MRI results are not reported in this manuscript and were used only as a way to identify the cortex which is activated during a stories listening task for all participants as reported previously (29, 49). There were no significant between-groups differences in cortical representation during stories listening vs. noise stimuli (49). Therefore, a joint activation map was constructed and used for subsequent functionally-constrained diffusion structural connectometry analyses, replicating our prior pilot work (Supplementary Figure 1) (31).

#### Diffusion Acquisition and Preprocessing

Multi-shell diffusion data were collected concurrently during the same structural MRI acquisitions with a DWI-SE sequence (b = 1000, 2000, 3000 with 30, 60, 120 directions, respectively; 10 b0, TR/TE = 4,296/168 ms, Matrix = 112 x 112, 72 slices, 2.0 × 2.0 × 2.0 mm resolution) with multiband (factor 4) and in-plane SENSE (factor 1.2) acceleration. All 3 shells took 15 min and 48 s to acquire. The b3000 scan was prioritized in the exam series, as the high b-value HARDI acquisition maximizes higher-order analytic flexibility, and lasted for 9 min and 25 s. For the purposes of this study, only the b3000 data were analyzed to maximize the number of participants in each group. Diffusion data were preprocessed using TORTOISE (50). Images were denoised (51); cleaned of Gibbs ringing artifact (52); and corrected for motion, eddy, and geometric distortions (50, 53). Original diffusion vectors were rotated at each relevant step in the processing pipeline. The T2-weighted scan that was used for rigidly registering the dMRI data and for the B-spline geometric distortion correction was first skull stripped using AFNI (45).

### Statistical Analyses

#### Analysis of Demographic and Neuropsychological Data

Group differences were evaluated using ANOVA for continuous variables (such as age at time of testing, gestational age at birth, and language scores) and Fisher's exact test for categorical variables (such as sex, race, ethnicity, parental education, and family income). A language composite was computed by averaging the PPVT4 and EVT2 standardized scores together to replicate our previous structural connectometry work (CELF-P scores were not included in the composite because they were not obtained for our pilot work) (31). Language composite scores were related to structural connectometry results as outlined below.

#### Diffusion Reconstruction and Connectometry Database Creation

After preprocessing, data were reformatted and imported to DSI Studio (http://dsi-studio.labsolver.org) for further analyses. All datasets underwent quality control, during which all diffusion volumes with a neighboring correlation coefficient of <0.9 were removed. Participants were excluded if more than 10% of volumes were removed. Diffusion data were then reconstructed using Q-Space Diffeomorphic Reconstruction (QSDR) (54, 55) to obtain each participant's spin distribution functions (SDFs) in a standard (MNI) space. Please see our previous work for a more detailed description of this methodology (31). Default parameters were used for QSDR (mean diffusion ration = 1.25).

#### Between Groups Analysis of Variance for Diffusion Imaging Data

An in-house extension of diffusion connectometry that allowed for an analysis of variance (ANOVA) among the three groups was conducted to obtain those SDFs that both met assumptions and had a statistically significant omnibus F (56, 57). First, an equal proportion of participants (50% from each of the three groups) were chosen from each group to create a study-specific template by averaging SDFs obtained from QSDR. To create the connectometry database, each subject's SDFs were then aligned to this newly created template, and a spatial correlation coefficient was calculated to assess the quality of the registration. Additionally, SDFs for each subject were assessed for outliers and any subject with a normalized average SDF value > 2 standard deviations above the rest of the group was excluded. No participants needed to be excluded at this step. SDF values for each local fiber direction were exported for the ANOVA.

**Figure 1:**
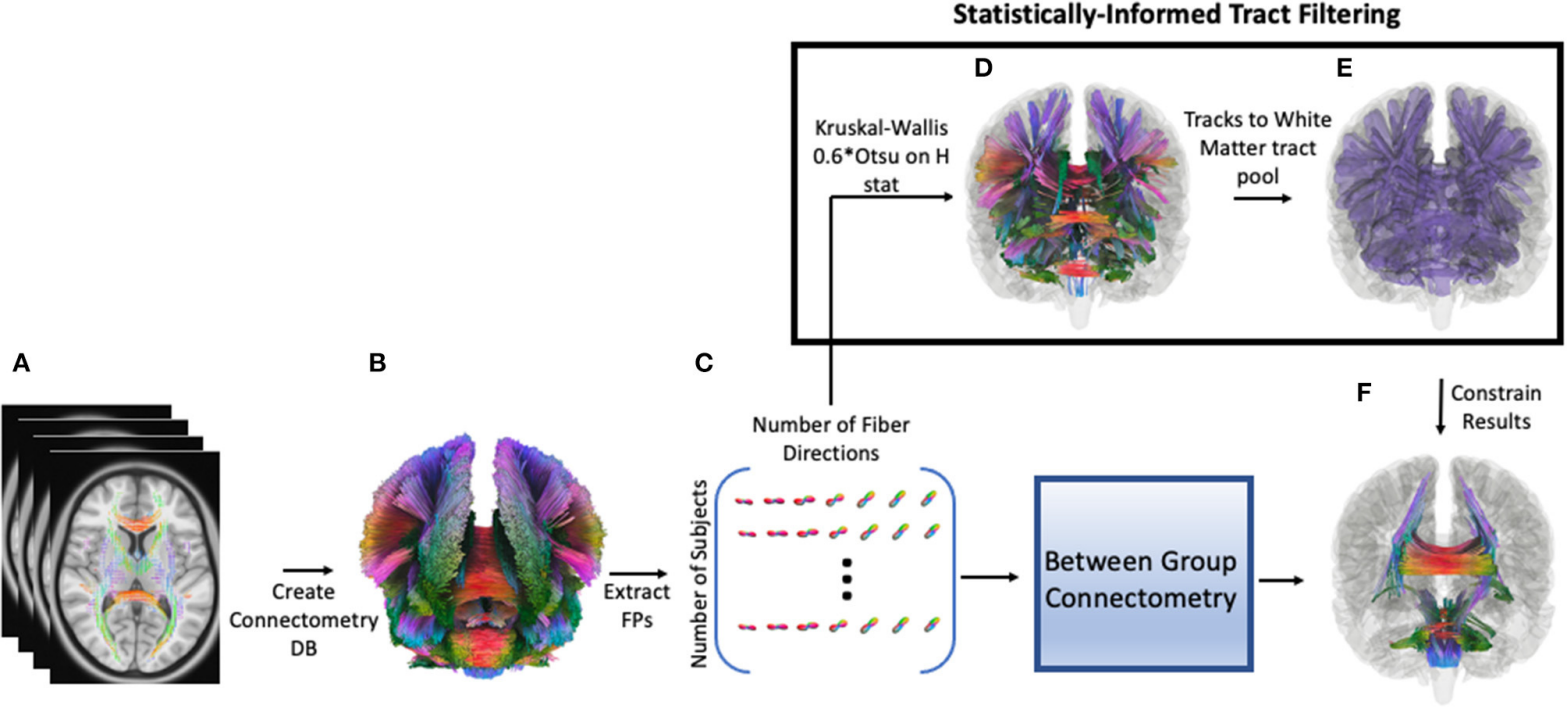
Pipeline for analysis. First, preprocessed diffusion data for each participant is reconstructed using QSDR, followed by creating a population template and subsequent connectometry database (DB, **A,B**). Then individual “local connectomes”–i.e., individual connectome fingerprints (FP)–are extracted from this database as a matrix, size of number of subjects by (voxels x local fiber directions, **C**). Each column represents the SDF value for an individual local fiber direction, which is used in a Kruskal-Wallis test (non-parametric ANOVA) to generate an H-statistic for that direction. A threshold is calculated for these H statistics (0.6*Otsu's threshold) and surviving fiber directions are clustered (20 voxels) and converted into a region of interest (ROI); the ROI is inverted so that this inverted mask can be used a terminative mask to generate a tract pool of tracts that survived the Kruskal-Wallis thresholding **(D)**. In other words, any track that terminates outside of the original ROI is excluded. These tract segments are converted to an ROI (statistically-informed tract filtering show in **E**) and used to filter the results from between-group connectometry analysis **(F)**.

Since there was no guarantee that SDFs are normally distributed across tracts (i.e., local connectomes), a non-parametric ANOVA (Kruskal-Wallis test) was performed among the three groups to obtain the omnibus H statistic. 0.6^*^Otsu's method (58) was used on the H statistics to determine which SDFs would be passed on to between group analyses using the connectometry framework. In this case, group connectometry between groups acted as a *post-hoc* comparison, which corrected for both spurious spatial correlations and multiple comparisons. Dataset manipulation, including Kruskal-Wallis testing and thresholding, was carried out using Python 3.9. To create the mask used in connectometry to restrict tracks to only those resolved by the Kruskal-Wallis, we (1) performed a cluster correction (family-wise error correction) of 20 mm, with the nearest neighbor method including faces, edges, and corners; (2) negated the resulting mask, so only the areas of the brain outside of the resulting clusters were masked; (3) used this negated mask as a terminative for whole-brain tractography using the connectometry database file (QA threshold = 0.6^*^Otsu's threshold, angular threshold = 60, 100000 seeds); and (4) converted the resulting tracks in an ROI that could be used to constrain results from between group comparisons. Figure 1 shows a schematic of the pipeline starting after QSDR reconstruction. Code to generate the data needed for connectometry can be found at https://github.com/willi3by/dsi-studio-anova.

#### Connectometry Analysis of Diffusion Data and Relation to Performance

Connectometry was performed between groups. The t-threshold was set to 2.0 for between group analyses, meaning only SDFs with a moderate to high correlation were used. Other parameters included a length threshold of 40 mm, 2 rounds of topology informed pruning of tracts, and FDR was calculated using 4,000 permutations and was set to 0.05. Quantitative anisotropy (QA) was normalized for the SDFs to account for any changes due to age, since there were not enough participants in each group to responsibly include this variable in the final model. Resulting tracks from this analysis were then constrained by the white matter pool generated from the Kruskal-Wallis analysis (Figure 1) serving as a statistical heuristic for informing track selection.

Within-group connectometry assessing white matter connectivity differences related to performance on standardized language assessments (the language composite) was carried out without the restriction of the Kruskal-Wallis test, since there was no comparison with other groups. WNV scores were included as a nuisance regressor in all within group analyses to control for general abilities. All connectometry parameters remained the same, but the t-threshold was increased to 2.5, as the results were not further constrained by a previous statistical test. This allowed for more specificity in the criteria for which local connectomes were selected (59). Within-group analyses were performed at the whole-brain level and within the fMRI-derived stories listening network (see functional MRI acquisition and joint activation map).

#### Analyses of Sex as a Biological Variable

Due to the previously reported impact of sex on prematurity and on language, sex was included in analyses as a biological variable (60–62). Group differences in sex distribution and differences in outcomes such as language scores and structural connectivity for females vs. males were investigated.

## Results

### Demographic and Neuropsychological Assessment

There were no significant group differences in age at time of testing, sex, race, ethnicity, handedness laterality quotient, combined family income, or highest level of parental education (Table 2). There were significant group differences in gestational age, as expected (omnibus ANOVA had a *p* < 0.001). The mean GA for the TC group was 39 weeks and 4 days, the mean for the EPT group was 26 weeks and 5 days, and the mean for the EPT-HLD group was 25 weeks and 5 days. *Post hoc* testing (Tukey's) revealed this was due to the EPT and the EPT-HLD groups having a significantly lower GA than the TC group, as anticipated. There were no statistically significant differences in GA between the EPT and EPT-HLD groups. There were no significant group differences in standardized scores for the PPVT4, EVT2, CELF-P, or WNV (Table 2).

**Table 2:** Demographics and neuropsychological data for entire sample.

		**EPT-HLD (*n* = 8)**	**EPT (*n* = 16)**	**TC (*n* = 28)**	***p*** **-value**
Age (years, mean ± SD)		5.89 ± 0.64	5.31 ± 1.02	5.65 ± 0.97	0.32
Gestational age (weeks + days)		25 + 5	26 + 5	39 + 4	<0.001
Sex	Females	4	9	14	0.93
	Males	4	7	14	
Race	White/Caucasian	3	10	18	0.57
	Black/African American	5	4	7	
	Other/multiple	0	1	2	
	No response	0	1	1	
Ethnicity	Hispanic/Latino/Latina	1	1	2	0.81
	Not Hispanic/Latino/Latina	7	15	26	
	No response	0	0	0	
Family income	< $50,000	4	5	9	0.82
	$50,000–$100,000	2	3	7	
	>$100,000	2	8	12	
	No response	0	0	0	
Parental education	High school	1	0	5	0.09
	College	4	10	7	
	Post graduate	3	6	16	
	No response	0	0	0	
Receptive language	PPVT-4 (Mean ± SD)	110 ± 14	111 ± 13	113 ± 14	0.71
Expressive language	EVT-2 (Mean ± SD)	99 ± 7	107 ± 11	110 ± 16	0.09
Language morphology	CELFP-WS (Mean ± SD)	9.43 ± 2	9.71 ± 2	10.46 ± 3	0.49
General abilities	WNV (Mean ± SD)	98 ± 14	103 ± 16	108 ± 14	0.17

*Categorical variables were tested using Fisher's Exact Test and p-values are reported. Continuous variables were tested using Analysis of Variance (ANOVA) tests and p-values are reported. EPT-HLD, Extremely Preterm with History of Language Delay/Disorder; EPT, Extremely Preterm without Language Delay or Deficit; TC, Term Comparison Children; SD, Standard Deviation; PPVT-4, Peabody Picture Vocabulary Test; EVT-2, Expressive Vocabulary Test; CELFP-WS, Clinical Evaluation of Language Fundamentals Preschool Word Structure Scaled Score; WNV, Wechsler Non-Verbal Scale of Ability*.

### Between-Groups Structural Connectometry

**Figure 2:**
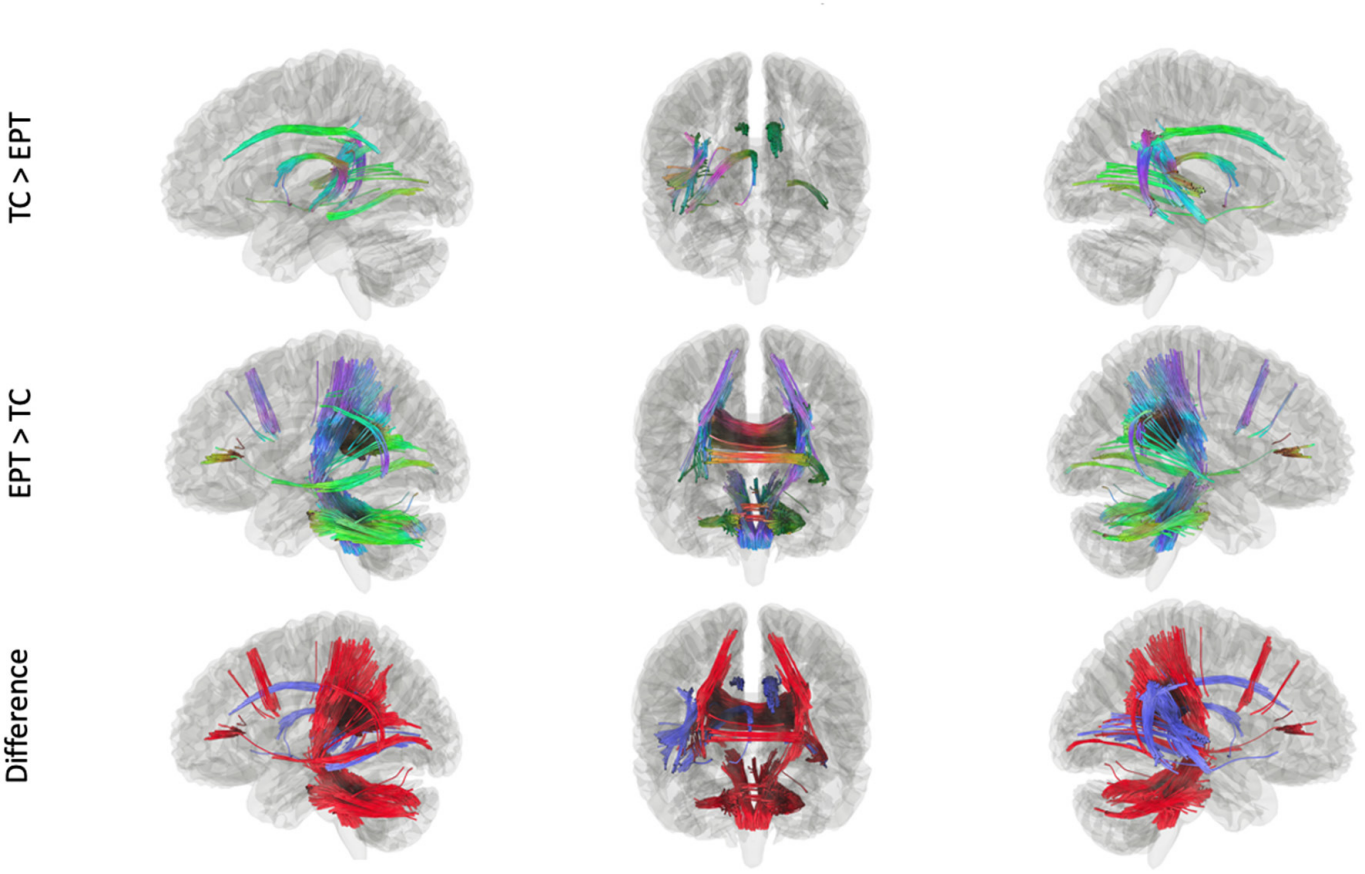
Tracks within the ANOVA (Kruskal-Wallis) white matter pool that were significant in *post-hoc* analyses. There were no tracks in which EPT-HLD had a significant difference with either EPT or TC (t = 2.0, length threshold = 20 voxels, FDR = 0.05, 4,000 permutations, 2 rounds of tract trimming). There were tracts in which TC > EPT (top row) and EPT > TC (middle row). The bottom row shows the difference in these results (blue = TC > EPT, red = EPT > TC).

In our Kruskal-Wallis analysis, 41.1% of SDFs passed the 0.6^*^Otsu's threshold on the H-statistic. After filtering by the tracks generated from the Kruskal-Wallis procedure, whole-brain results showed significant differences between TC and EPT. Tracks in which TC had greater connectivity than EPT include the body of the corpus callosum, bilateral cingula, right fornix, right arcuate fasciculus, and middle segments of the inferior fronto-occipital fasciculus (IFOF) (Figure 2; top panel). Tracks in which EPT had greater connectivity than TC include bilateral corticospinal tracts (CST), splenium and genu of the corpus callosum, bilateral posterior arcuate fasciculi, middle cerebellar peduncle, and additional cerebellar white matter (Figure 2, middle panel). There were no tracks in which there were significant differences between EPT-HLD and either TC or EPT. There were no tracks—after filtering by the Kruskal-Wallis procedure—that were statistically significant between groups when constrained by the fMRI stories network.

### Within Groups Structural Connectometry

#### Connectometry Within Groups: Constrained to Functional Stories Network

**Figure 3:**
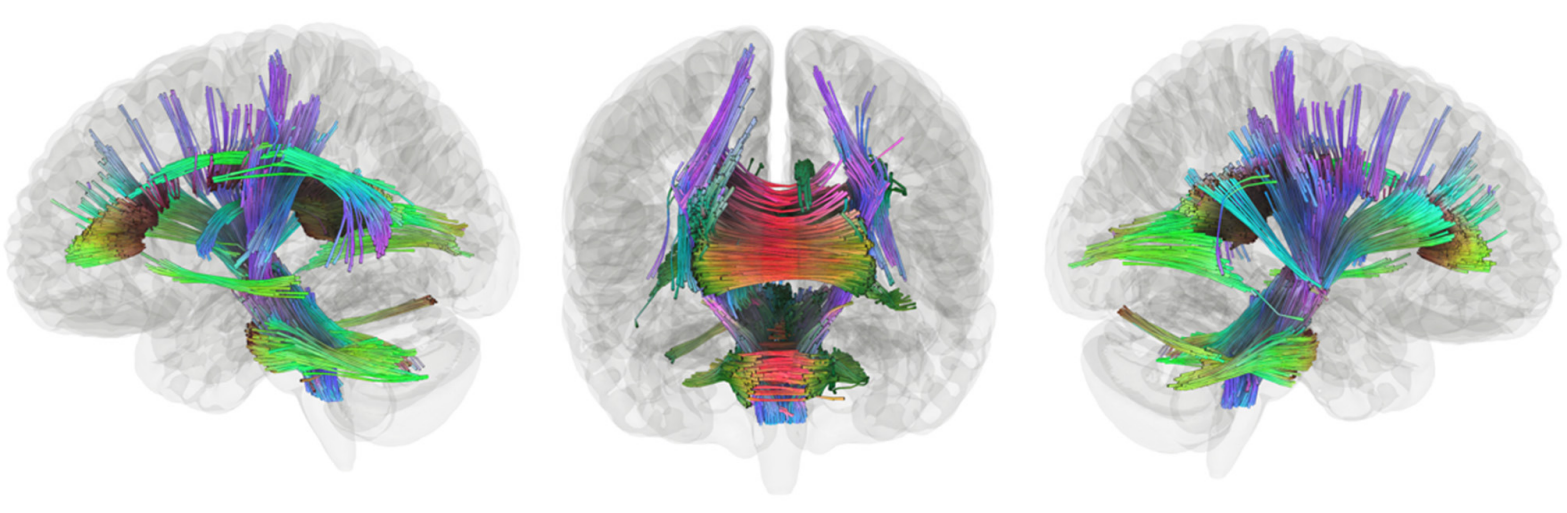
Tracks positively associated with composite language performance within TC group. Tracts that were positively correlated with language performance, controlling for WNV, within TC (t = 2.5, length threshold = 20 voxels, FDR = 0.05, 4,000 permutations, 2 rounds of tract trimming). Results include bilateral corticospinal tract, much of the corpus callosum, middle cerebellar peduncle, left arcuate fasciculus, left inferior longitudinal fasciculus, and left cingulum.

**Figure 4:**
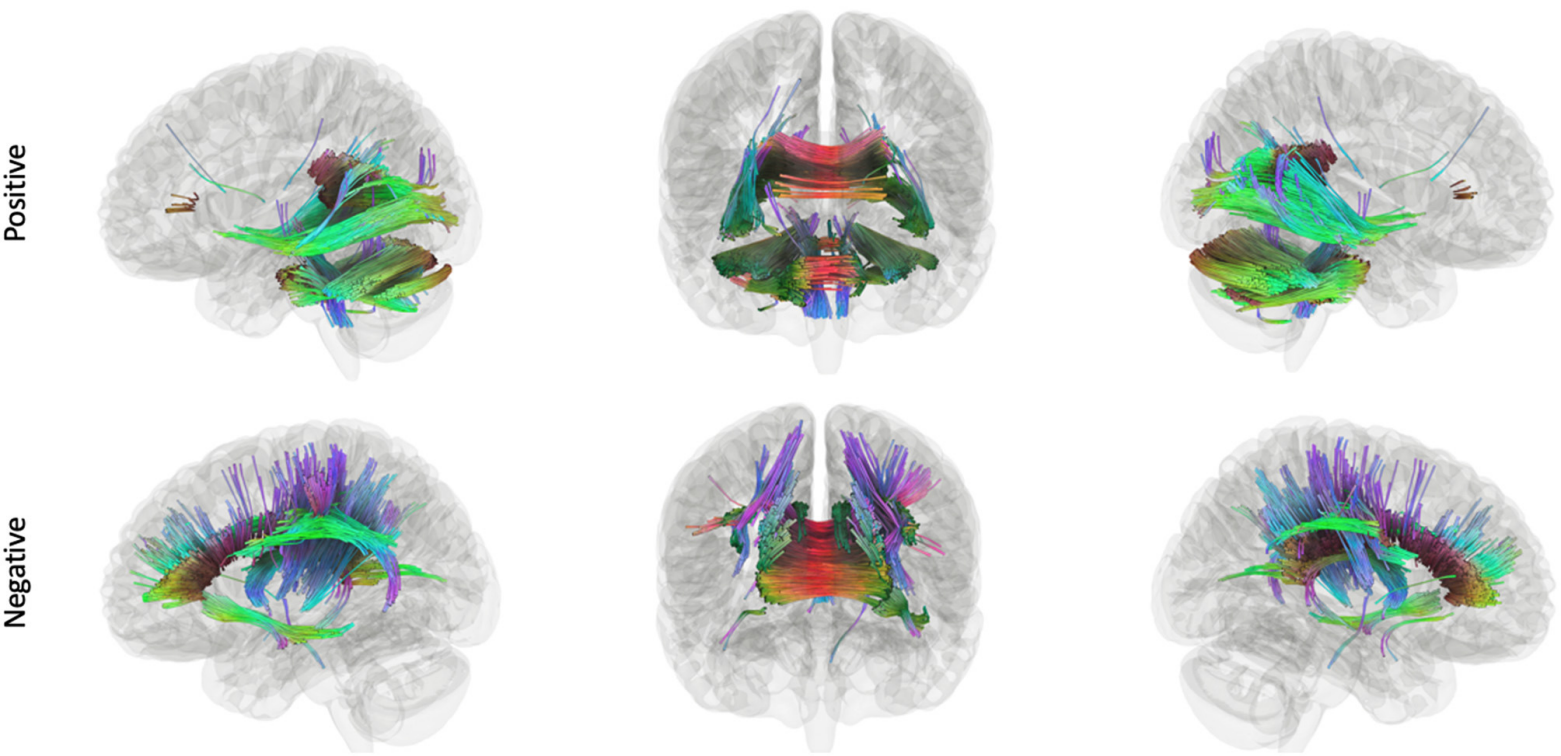
Tracks positively and negatively associated with composite language performance within the EPT group. Tracts that were positively (top row) and negatively (bottom row) correlated with language performance, controlling for WNV, within EPT (t = 2.5, length threshold = 20 voxels, FDR = 0.05, 4,000 permutations, 2 rounds of tract trimming). There is a clear dichotomy of superior tracts traditionally correlated with language (left arcuate fasciculus, corpus callosum, left lateral fronto-occipital fasciculus, etc.) that are negatively correlated in EPT. Inferior and posterior tracts that are not typically associated with language (posterior inferior fronto-occipital fasciculus, cerebellum, cerebellar peduncles, splenium) that are positively associated with language performance.

Connectometry in the stories network within TC revealed a positive association between connectivity and performance in the white matter pathways including the bilateral corticospinal tracts, much of the corpus callosum, the middle cerebellar peduncle, the left arcuate fasciculus, the left inferior longitudinal fasciculus, and the left cingulum (Figure 3). Within the EPT-HLD group, when constrained to the functional stories network, analyses showed a positive association between white matter connectivity and performance in the bilateral corticospinal tracts, much of the corpus callosum, the middle cerebellar peduncle, the left arcuate fasciculus, the left inferior longitudinal fasciculus, and the left cingulum (Supplementary Figure 2). Thus, the white matter pathways positively associated with language scores for the group of EPT-HLD children very closely resembles that of the TC group. Within the well-performing EPT group, when constrained to the stories network, analyses revealed some mixed results (Figure 4). There is a clear dichotomy of superior tracks traditionally correlated with language (including the left arcuate fasciculus, corpus callosum, and left lateral fronto-occipital fasciculus) that were negatively correlated with language scores for the EPT group in this analysis. Inferior and posterior tracts that are not typically associated with language (such as the posterior inferior fronto-occipital fasciculus, cerebellar tracts, cerebellar peduncles, and splenium) were positively associated with standardized language performance for the EPT group.

#### Connectometry Within Groups: Whole-Brain

The stories listening network was very widespread and results were nearly identical to whole-brain analyses. Therefore, we only reported results within the stories listening network for clarity. Whole-brain results are available upon request.

### Analyses of Sex as a Biological Variable

There were no significant differences between groups in the distribution of males vs. females (Table 2). There were no significant differences between males vs. females in standardized neurobehavioral scores (PPVT4, EVT2, WNV, CELF-P) or in gestational age (GA). There were significant differences in structural connectometry findings between males and females. These results are presented in the Supplementary Figure 3. Within the TC and EPT groups, males generally had greater connectivity than females (shown in red). However, females in the EPT and TC groups appeared to have increased cerebellar connectivity (shown in blue). There were no significant tracks within the EPT-HLD group or across the entire cohort (TC+EPT+EPT-HLD).

## Discussion

Our first testable hypothesis was that well-performing EPT children with no history of brain injury, language delay, or neurological disorder would have increased extracallosal structural connectivity, including some of the cerebellar white matter, vs. their TC counterparts at 4–6 years of age. The data support this hypothesis, validating findings we previously reported in a smaller, distinct group of children born EPT (31). In our *post-hoc* analyses, significant differences between the EPT, EPT-HLD, and TC groups appear to be driven by differential structural connectivity in the EPT and TC groups. Tracts in which TC had greater connectivity than EPT include tracts traditionally associated with the canonical language network, such as the arcuate fasciculus (Figure 2, top panel). Tracts in which EPT had greater connectivity than TC include bilateral posterior white matter areas, including the cerebellum (Figure 2, middle panel). There were no tracts in which there were significant differences between EPT-HLD and either TC or EPT. In this regard, we replicate our earlier work (31). Findings are consistent with recent reports of decreased structural connectivity and/or decreased fractional anisotropy in body of the corpus callosum and white matter of the left hemisphere in preterm children vs. term children, as our EPT participants relay on an atypical pattern of tracks to connect the right and left temporal regions (including the splenium and cerebellum) (41, 63, 64). These differential findings are also consistent with reports of increased functional connectivity in EPT children vs. TC, including our own work in this same cohort of children (29, 30, 42, 43, 49). Thus, while connectivity in some brain areas–including periventricular areas known to be susceptible to the insults of extreme prematurity, such as the corpus callosum–is reduced in prematurity, increased extracallosal structural connectivity seems to support the increased bitemporal interhemispheric functional connectivity we have reported in this cohort of preterm children as they perform language tasks, and which others have reported in their own distinct groups of preterm children (29, 30, 49, 65, 66).

The second hypothesis we tested was that the EPT-HLD group would have lower scores on standardized language assessments than their well-performing EPT counterparts and would lack the extracallosal hyperconnectivity we previously reported for the well-performing EPT group vs. their term counterparts (TC). We failed to reject the null hypothesis in that there were no significant differences in performance for the EPT, EPT-HLD, and TC groups on standardized assessments at 4–6 years of age. Furthermore, while our data do seem to support the hypothesis that the EPT-HLD group would lack statistically significant hyperconnectivity in extracallosal regions compared to the EPT group, this could be due to a lack of sufficient power. We observed a non-significant relationship (results not shown) in which the EPT-HLD group had patterns of increased connectivity vs. the EPT group in anterior white matter and callosal areas and decreased connectivity vs. the EPT group in posterior and cerebellar areas (similar to the TC group). This warrants further investigation in a larger cohort of extremely preterm children with a history of language delay or disorder.

**Figure 5:**
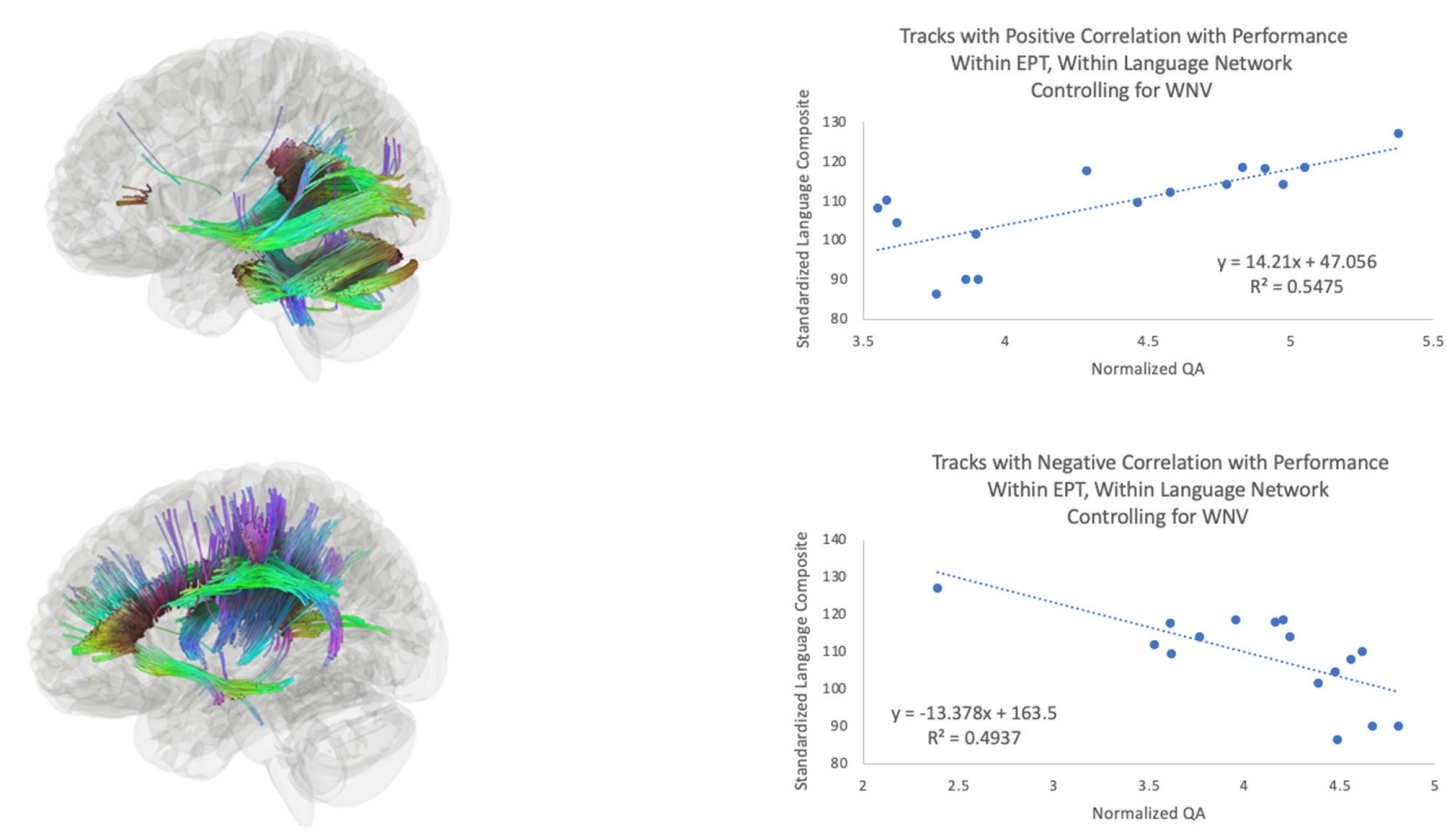
Tracks positively associated (top) and negatively associated (bottom) with composite language performance within the EPT group. Scatterplots of language performance by normalized QA value within the tracts that were significant for the EPT group (see Figure 4) within the language network, for the analysis of the effects of language performance on white matter connectivity, controlling for WNV.

The third hypothesis we tested was that extracallosal structural connectivity, including that of the cerebellar white matter, would be positively associated with language scores for the EPT and EPT-HLD groups, but not for the TC group. Whole-brain connectometry within TC revealed positive correlations between performance and white matter connectivity in much of the white matter in the brain, including bilateral corticospinal tracts, the corpus callosum, the middle cerebellar peduncles, the left arcuate fasciculus, the left inferior longitudinal fasciculus, and the left cingulum (Figure 3). In the EPT-HLD group, the findings were similar to the TC group (Supplementary Figure 2) in that much of the white matter had a positive association with performance, including the corticospinal tracts, corpus callosum, middle cerebellar peduncles, left arcuate fasciculus, left inferior longitudinal fasciculus, and left cingulum. No tracks had a negative association. Conversely, in the well-performing EPT group, tracks positively associated with performance included the bilateral posterior inferior fronto-occipital fasciculi, bilateral cerebellar white matter, bilateral cerebellar peduncles, and the splenium (Figure 4). Normalized QA in these posterior and cerebellar white matter pathways accounted for 55% of the variance in the standardized language composite scores for the EPT group specifically (Figure 5). Tracks negatively associated with performance for the EPT group specifically included pathways traditionally thought to be connecting canonical language areas (67, 68), including the left arcuate fasciculus, much of the corpus callosum, and the left lateral fronto-occipital fasciculus (Figure 4). Normalized QA in these pathways accounted for 49% of the variance in language composite scores for the EPT group exclusively (Figure 5). A comparison of positive and negative associations (Figure 5) shows a distinct dichotomy that suggests greater connectivity in superior, anterior tracts leads to worse performance while greater connectivity in inferior, posterior tracts leads to better performance. For the EPT group specifically, cerebellar pathways appear preferentially related to language performance over other periventricular areas such as the corpus callosum. Indeed, one might speculate that, for children born near the end of the second trimester of gestation, it is not only a positive adaptation to recruit cerebellar pathways to support language but also a maladaptive response to continue to rely on canonical language networks, such as the arcuate fasciculus. Recent work from our group has shown that anterior tracts support language in normal development while posterior tracts support non-verbal functions (69). The results we report in this paper suggest that well-performing EPT children employ alternate mechanisms to attain functioning within normal limits. This is consistent with our previously reported work in a distinct, non-overlapping group of EPT children (31).

These results are congruent with what is known about the developing preterm brain. The last trimester is a period of rapid and intense brain growth, including synaptogenesis, expansion of white and gray matter volume, cortical folding, and axonal growth and myelination in the cerebrum and cerebellum (70, 71). All of these processes are impacted by prematurity, as the fetus experiences this critical period of development ex-utero (71). Heterogeneity is a hallmark not only of the etiology and clinical sequelae of preterm birth but also of premature brain development (2). While greater atypicality on structural imaging (e.g., decreased FA in the cerebellum) has been associated with poor cognitive and language outcomes, there is high variability between preterm infants and preterm children in the spatial location of this atypicality (64, 72–74). However, periventricular white matter seems particularly vulnerable to injury and dysmaturation (71). In a cohort of children imaged from 25 to 45 weeks of gestation, local connectivity of structures such as the cerebellum, thalamus, and cingulum was impacted by the degree of prematurity (63). The cerebellum, a structure that is developing faster than any other brain region during the last trimester of gestation during which our participants were facing challenges ex-utero in the NICU, is being increasingly recognized as playing an important role in language development (31, 43, 75–77). Our reported results strengthen this conclusion and highlight the need for directed investigation into cerebellar development and connectivity in prematurely born individuals.

Of note, for the EPT-HLD group, white matter pathways positively associated with language scores were quite similar to that of our TC group (e.g., anterior and callosal in nature). Every participant in the EPT-HLD group received speech and language therapy by a pediatric speech language pathologist after diagnosis of language delay or disorder. We cannot speculate what the structural connectivity profiles of these children were like at 2 years of age when most of them were diagnosed with a mixed receptive-expressive language impairment. Thus, we cannot determine if this is a marker of a positive response to therapy or if this is a phenotype which required speech and language therapy to overcome and attain typical language function. Future, prospective studies will be needed to determine the predictive power of structural connectometry for preterm children with and without language delays or disorders.

We included sex as a biological variable in our analyses across and within groups. While there were no significant differences in performance by sex, there were significant differences in structural connectivity, with males having increased connectivity versus females as a rule. This might seem surprising, given the increased risk for neurodevelopmental impairment associated with male sex; however, this is consistent with other structural imaging studies in preterm children that have suggested prematurity disrupts the differential impact of sex on structural connectivity in typically-developing term-born children (78).

Our study has a few key limitations. The most significant limitation is our small sample size included in the final analyses, particularly for the EPT-HLD group. This might limit our power to identify structures or group differences in connectivity that have associations with language performance. Despite this, we feel the associations we report are likely to be the most robust findings (as evidenced by the replication of our previous pilot work). Future work will expand our study group and follow these children longitudinally. Another limitation is that our children with a history of diagnosed language delay or deficit are now performing within normal limits on standardized assessments of language (in contradiction to our initial hypothesis) despite being diagnosed by both medical provider and SLP with language delay, deficit, or disorder. This is congruent with recent work indicating most impairment in children born EPT improves over time (17). Furthermore, we feel that the history of a diagnosis of language deficit is clinically meaningful not only to providers but also to these children and their families. Our results might signify a marker of resiliency in language functioning in the context of prematurity or might serve as a marker of response to therapy. This represents an exciting avenue of future scientific inquiry, and further studies are needed to determine the relative resilience of these brain networks over time. We believe this study, while cross sectional, contributes valuable information to this research question which is congruent with the existing body of literature. Finally, our implementation of ANOVA within DSI Studio to investigate differences in structural connectometry between 3 groups is novel and—to our knowledge—has not been implemented before. While we feel this is a strength of our approach and will prove useful to other scientists employing structural connectometry methods to elucidate not only white matter diffusivity but also density of connections, it is true that future studies will be needed to demonstrate its generalizability and replicability.

In conclusion, we employ novel state-of-the-art higher-order tensor-free structural connectometry methods to interrogate differences in connectivity between children born EPT with no history of language issues, EPT children with a history of language delay or deficit, and term comparisons. We report differential associations between white matter connectivity and language performance between groups, suggesting that greater connectivity in superior, anterior tracts leads to worse performance for EPT children while greater connectivity in inferior, posterior tracts leads to better performance. Importantly, for the EPT group specifically, cerebellar pathways appear preferentially related to language performance over other periventricular areas such as the corpus callosum. We demonstrate that posterior and cerebellar structural connectivity supporting functional hyperconnectivity is a robust correlate of language functioning in 4–6-year-old children born EPT. Future work will follow these children longitudinally to investigate the impact of environmental factors on developing language networks and resiliency in the preterm brain.

## Data Availability Statement

The raw data supporting the conclusions of this article will be made available by the authors, upon reasonable request and might require a data use agreement.

## Ethics Statement

The studies involving human participants were reviewed and approved by Institutional Review Boards (IRB) of Cincinnati Children's Hospital Medical Center, the Perinatal Scientific Review Committee of the TriHealth medical system, and the University of Cincinnati. Written informed consent to participate in this study was provided by the participants' legal guardian/next of kin.

## Author Contributions

MB-D: conceptualization, resources, data acquisition, data curation, formal analysis, funding acquisition, investigation, methodology, project administration, roles/writing—original draft, and writing—review and editing. BW: data curation, data visualization, formal analysis, investigation, methodology, roles/writing—original draft, and writing—review and editing. SM and NP: conceptualization, resources, funding acquisition, investigation, methodology, supervision, and writing—review and editing. UN: data acquisition, data curation, and writing—review and editing. DK: conceptualization, resources, data curation, formal analysis, funding acquisition, investigation, methodology, project administration, supervision, and writing—review and editing. All authors contributed to the article and approved the submitted version.

## Funding

This work was funded by an award from the National Institute of Child Health and Human Development (K12-HD028827 for MB-D), an award from the National Center for Advancing Translational Sciences (KL2-TR001426 for SM), an awards from the National Institute of Neurological Disorders and Stroke (K23-NS117734 for MB-D, R01-NS094200 and R01-NS096037 for NP), and a Procter Scholar Award (MB-D) from the Cincinnati Children's Research Foundation.

## Conflict of Interest

The authors declare that the research was conducted in the absence of any commercial or financial relationships that could be construed as a potential conflict of interest.

## Publisher's Note

All claims expressed in this article are solely those of the authors and do not necessarily represent those of their affiliated organizations, or those of the publisher, the editors and the reviewers. Any product that may be evaluated in this article, or claim that may be made by its manufacturer, is not guaranteed or endorsed by the publisher.
