# Supplementary material for: Extracallosal Structural Connectivity Is Positively Associated With Language Performance in Well-Performing Children Born Extremely Preterm
Source: Front Pediatr. 2022 Mar 18;10:821121. doi: 10.3389/fped.2022.821121 (PMC8971711; doi:10.3389/fped.2022.821121)

SUPPLEMENTARY MATERIAL

Supplementary Figure 1. Tracks resolved in the template used for connectometry when using the stories listening language network as a region of interest (ROI, pink) to functionally constrain connectometry. The ROI is derived from the joint activation map obtained while all participants were participating in a passive stories listening task in functional MRI (see Barnes-Davis, et al., 2021, for further details).

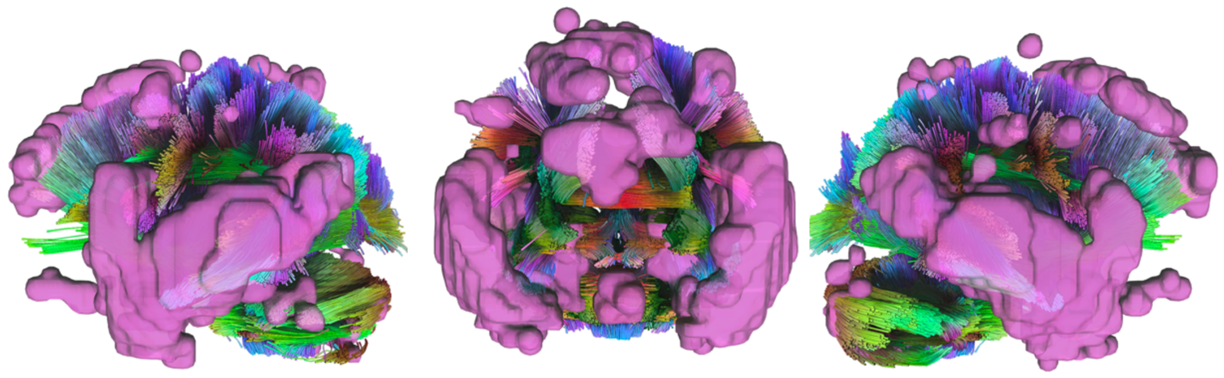

Supplementary Figure 2. Tracks that were positively correlated with language performance, controlling for WNV, within EPT-HLD (t=2.5, length threshold=20 voxels, FDR=0.3, 4000 permutations, 2 rounds of track trimming). Results include bilateral corticospinal tracts, much of the corpus callosum, middle cerebellar peduncle, left arcuate fasciculus, left inferior longitudinal fasciculus, and left cingulum, and are very similar to results in TC. Future studies will explore this finding in larger cohorts.

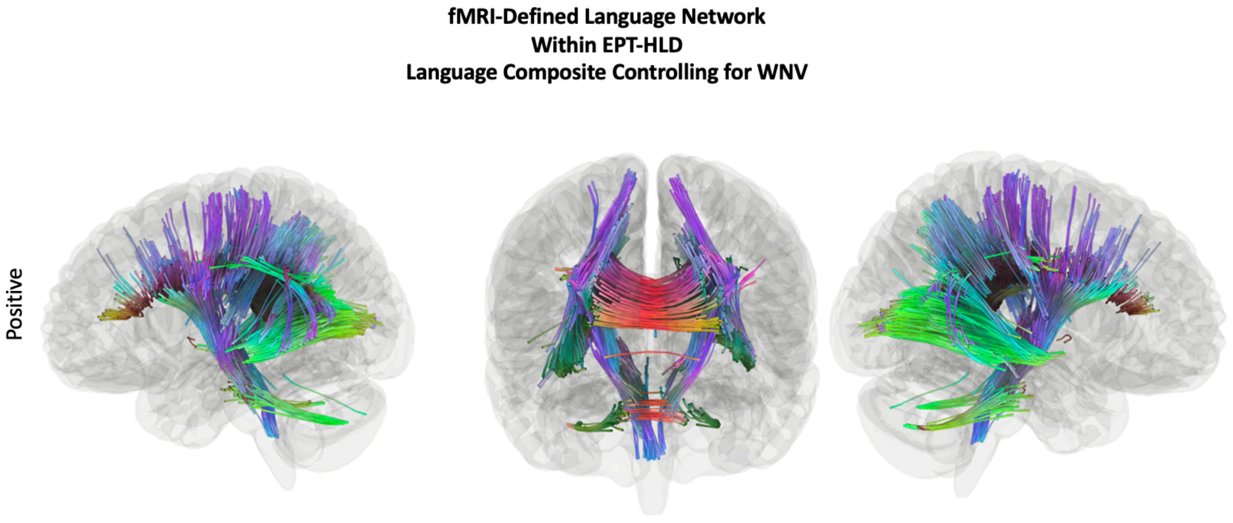

Supplementary Figure 3. Tracks with a significant relationship to sex within TC and EPT. There were no significant tracks in EPT-HLD or over the entire cohort. Results

show tracks in which males had greater connectivity than females (red) and those in which females had greater connectivity than males (blue).

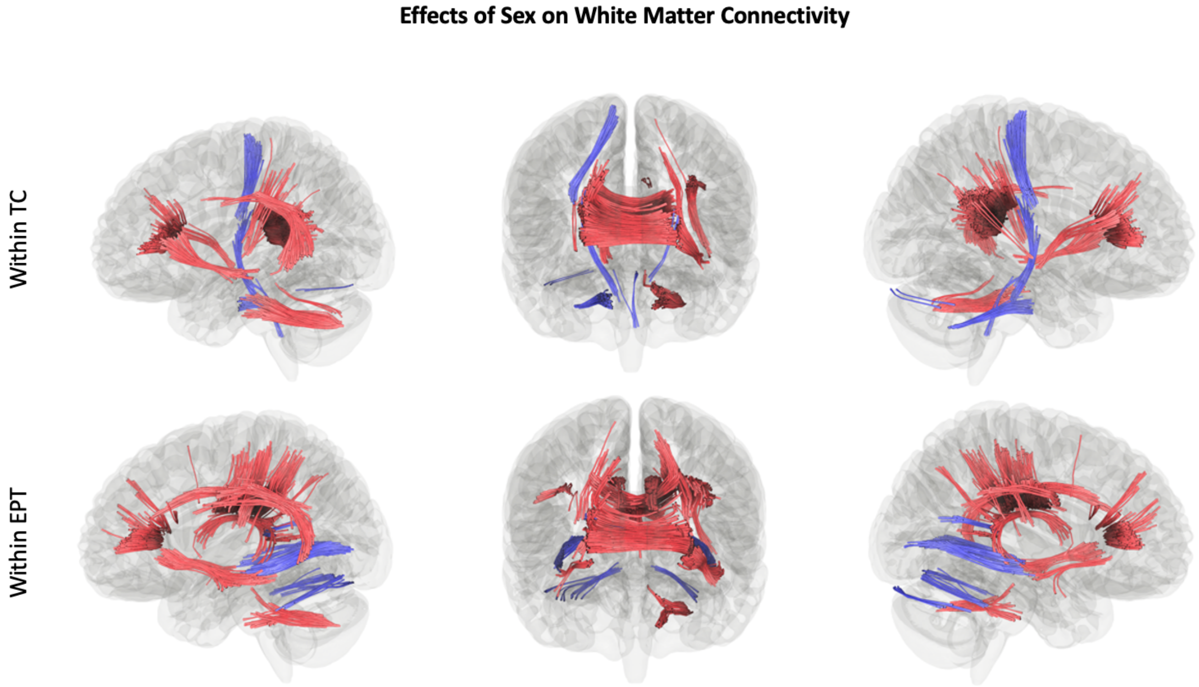

Supplement: Supplementary file 1 [file Data_Sheet_1.PDF]
